# Two-year imaging outcomes from a phase 3 randomized trial of secukinumab in patients with non-radiographic axial spondyloarthritis

**DOI:** 10.1186/s13075-023-03051-5

**Published:** 2023-05-16

**Authors:** Juergen Braun, Ricardo Blanco, Helena Marzo-Ortega, Lianne S. Gensler, Filip Van den Bosch, Stephen Hall, Hideto Kameda, Denis Poddubnyy, Marleen van de Sande, Désirée van der Heijde, Tingting Zhuang, Anna Stefanska, Aimee Readie, Hanno B. Richards, Atul Deodhar

**Affiliations:** 1 Department of Rheumatology, Ruhr-University Bochum, Bochum, Germany; 2 Rheuma Praxis, Berlin, Germany; 3 Division of Rheumatology, Hospital Universitario Marqués de Valdecilla, IDIVAL, Santander, Spain; 4 NIHR Leeds Biomedical Research Centre, Leeds Teaching Hospitals NHS Trust, LIRMM, University of Leeds, Leeds, UK; 5 University of California, San Francisco, San Francisco, CA USA; 6 Department of Internal Medicine and Pediatrics, Ghent University, Ghent, Belgium; 7 VIB Center for Inflammation Research, Ghent, Belgium; 8 Department of Medicine, Monash University, Melbourne, Australia; 9 Toho University, Tokyo, Japan; 10 German Rheumatism Research Centre, Charité - Universitätsmedizin Berlin, Berlin, Germany; 11 Amsterdam Rheumatology and Immunology Center, Amsterdam UMC, University of Amsterdam, Amsterdam, Netherlands; 12 Leiden University Medical Center, Leiden, The Netherlands; 13 Novartis Pharmaceuticals Corporation, East Hanover, NJ USA; 14 Novartis Ireland Limited, Dublin, Ireland; 15 Novartis Pharma AG, Basel, Switzerland; 16 Oregon Health & Science University, Division of Arthritis and Rheumatic Diseases, Portland, USA

**Keywords:** Axial spondyloarthritis, Non-radiographic axial spondyloarthritis, Nr-axSpA, Secukinumab, X-ray, Radiograph, Imaging

## Abstract

**Background:**

Radiographic progression and course of inflammation over 2 years in patients with non-radiographic axial spondyloarthritis (nr-axSpA) from the phase 3, randomized, PREVENT study are reported here.

**Methods:**

In the PREVENT study, adult patients fulfilling the Assessment of SpondyloArthritis International Society classification criteria for nr-axSpA with elevated CRP and/or MRI inflammation received secukinumab 150 mg or placebo. All patients received open-label secukinumab from week 52 onward. Sacroiliac (SI) joint and spinal radiographs were scored using the modified New York (mNY) grading (total sacroiliitis score; range, 0–8) and modified Stoke Ankylosing Spondylitis Spine Score (mSASSS; range, 0–72), respectively. SI joint bone marrow edema (BME) was assessed using the Berlin Active Inflammatory Lesions Scoring (0–24) and spinal MRI using the Berlin modification of the AS spine MRI (ASspiMRI) scoring (0–69).

**Results:**

Overall, 78.9% (438/555) of patients completed week 104 of the study. Over 2 years, minimal changes were observed in total radiographic SI joint scores (mean [SD] change, − 0.04 [0.49] and 0.04 [0.36]) and mSASSS scores (0.04 [0.47] and 0.07 [0.36]) in the secukinumab and placebo-secukinumab groups. Most of the patients showed no structural progression (increase ≤ smallest detectable change) in SI joint score (87.7% and 85.6%) and mSASSS score (97.5% and 97.1%) in the secukinumab and placebo-secukinumab groups. Only 3.3% (*n* = 7) and 2.9% (*n* = 3) of patients in the secukinumab and placebo-secukinumab groups, respectively, who were mNY-negative at baseline were scored as mNY-positive at week 104. Overall, 1.7% and 3.4% of patients with no syndesmophytes at baseline in the secukinumab and placebo-secukinumab group, respectively, developed ≥ 1 new syndesmophyte over 2 years. Reduction in SI joint BME observed at week 16 with secukinumab (mean [SD], − 1.23 [2.81] vs − 0.37 [1.90] with placebo) was sustained through week 104 (− 1.73 [3.49]). Spinal inflammation on MRI was low at baseline (mean score, 0.82 and 1.07 in the secukinumab and placebo groups, respectively) and remained low (mean score, 0.56 at week 104).

**Conclusion:**

Structural damage was low at baseline and most patients showed no radiographic progression in SI joints and spine over 2 years in the secukinumab and placebo-secukinumab groups. Secukinumab reduced SI joint inflammation, which was sustained over 2 years.

**Trial registration:**

ClinicalTrials.gov, NCT02696031.

**Supplementary Information:**

The online version contains supplementary material available at 10.1186/s13075-023-03051-5.

## Background

Axial spondyloarthritis (axSpA) is a chronic inflammatory disease of the axial skeleton predominantly affecting the spine and sacroiliac (SI) joints [1]. Based on the presence or absence of definite structural changes in the SI joints on conventional radiographs, patients with axSpA are classified into 2 subtypes [2]. Patients with radiographic evidence of sacroiliitis fulfilling the modified New York (mNY) criteria are classified as having radiographic axSpA (r-axSpA, also known as ankylosing spondylitis [AS]) whereas patients who do not meet the mNY criteria but may show evidence of sacroiliitis on magnetic resonance imaging (MRI) are classified as having non-radiographic axSpA (nr-axSpA) [2–5]. Patients with nr-axSpA have largely similar levels of disease activity, pain, and health-related quality of life impairment as patients with r-axSpA [6, 7]. It has been reported that about 10 to 40% of patients with nr-axSpA progress to r-axSpA over a period of 2 to 10 years [8]. The key goals of the treatment of axSpA include improving symptoms, decreasing inflammation, improving function and quality of life, and preventing irreversible skeletal damage, e.g., new bone formation in the spine [9].

Secukinumab, a human monoclonal immunoglobulin (IgG1) antibody that selectively neutralizes human interleukin (IL)-17A, demonstrated rapid and sustained improvement in the signs and symptoms of AS in long-term treatment (up to 5 years) in the phase 3 MEASURE studies [10–13]. PREVENT was the first randomized placebo-controlled phase 3 study to evaluate the efficacy and safety of secukinumab in patients with nr-axSpA with objective signs of inflammation [14]. Secukinumab improved signs and symptoms throughout the study period. The primary endpoint of 40% improvement in the Assessment of SpondyloArthritis International Society (ASAS40) criteria was met [14]. Radiographic progression and the course of inflammation as assessed by conventional radiographs and magnetic resonance imaging (MRI) of SI joint and spine, over 2 years from the PREVENT study, are reported here.

## Methods

### Study design and patients

Study design and eligibility criteria have been reported previously in detail [14]. Briefly, PREVENT (NCT02696031) was a randomized, double-blind, placebo-controlled, phase 3 study in patients with nr-axSpA (Additional file 1: Fig. S1). Adult patients (aged ≥ 18) with a clinical diagnosis of nr-axSpA who met the ASAS classification criteria for axSpA and had objective signs of inflammation (MRI with SI joint inflammation and/or high-sensitivity C-reactive protein levels greater than the upper limit of normal) were included. Patients with radiographic evidence of sacroiliitis according to the mNY criteria as assessed centrally at screening (single read for eligibility) were excluded. Eligible patients were randomized (1:1:1) to receive subcutaneous secukinumab 150 mg with a loading dose, 150 mg without a loading dose, or placebo at baseline and weeks 1, 2, and 3, followed by monthly dosing starting at week 4. The secukinumab 150 mg non-loading dose group received placebo at weeks 1, 2, and 3 to maintain blinding. In case of inadequate response (based on clinical judgment of disease activity by the investigator and the patient), patients were allowed to be switched to open-label secukinumab or standard of care after week 20. All patients (except those who switched to standard of care) received open-label secukinumab 150 mg starting at week 52.

The study was conducted in accordance with the Good Clinical Practice guidelines and the Declaration of Helsinki and was approved by institutional review boards or independent ethics committees at each participating center. Written informed consent was obtained from all enrolled patients.

### Assessments

In this exploratory analysis, radiographic progression was assessed by conventional radiography and the course of inflammation by MRI, of SI joints and spine, over 2 years. Radiographs of the spine and SI joints were obtained at baseline and at week 104. MRI images of the spine and SI joints were obtained at baseline and weeks 16, 52, and 104.

#### Radiographs: SI joint

SI joint radiographs were scored according to the mNY grading method (grade 0: normal findings; grade 1: suspicious changes; grade 2: minimum abnormality with some sclerosis, minimal erosion, no marked joint space narrowing; grade 3: unequivocal abnormality—moderate or advanced sacroiliitis with erosion, sclerosis, widening, narrowing, and/or partial joint fusion [ankylosis]; grade 4: severe abnormality [total ankylosis]) [5]. The total sacroiliitis score was computed as a sum of scores for the left and right SI joints (range, 0–8). A mNY assessment grade ≥ 2 bilaterally or ≥ 3 unilaterally was considered mNY-positive. When 1 SI joint assessment grade was < 3 and the other SI joint was not evaluable, the image was considered not evaluable.

#### Radiographs: spine

Spinal radiographs were scored using the modified Stoke AS Spine Score (mSASSS) [15]. The mSASSS scores each vertebral unit 0 to 3 (0: no abnormality; 1: erosion, sclerosis, or squaring; 2: syndesmophyte; and 3: bridging syndesmophyte) [16]. Total mSASSS ranges from 0 to 72. The number of patients who developed new syndesmophytes after 2 years was also evaluated. A syndesmophyte was defined as a score of ≥ 2 for any individual vertebral corner within evaluable vertebral units. A new syndesmophyte was defined as an individual vertebral corner with a score of 0 or 1 at screening that changed to a score of 2 or 3 at week 104.

#### MRI: SI joint

SI joint bone marrow edema (BME) was assessed according to the Berlin Active Inflammatory Lesions Scoring (0–24).

#### MRI: spine

Spinal MRI images were assessed for signs of inflammation using the Berlin modification of the AS spine MRI (ASspiMRI) scoring (0–69) [17].

### Reading of images

At the final reading session (week 104), all baseline and post-baseline images were scored by 2 central independent and experienced readers who were blinded to treatment assignment and image sequence. The cases with the highest between-reader differences for change in score from baseline were identified for adjudication review by a third independent reader. Adjudication review was triggered independently for the spine and SI joint and each imaging modality. The top 5% of radiograph reads with the highest difference in change of scores, the top 5% of discrepant cases of total SI joint edema score, and the top 10% of discrepant cases of total spine edema scores were scored independently by a third reader. All data are reported from the final reading session (all time points up to week 104), which was independent from previous reading sessions (performed for eligibility assessment or for primary results analysis) [14].

### Statistical methods

The change from baseline in the total sacroiliitis score and mSASSS score was calculated based on the average of scores assigned by 2 or 3 readers (if available) and presented as a cumulative probability of change from baseline. For binary variables (mNY criteria and syndesmophyte status), the analysis was carried out using a single-reader approach or 2-reader agreement approach. The single-reader approach considered a patient to be mNY criteria-positive when assessed mNY-positive by at least 1 reader. Correspondingly, if at least 1 reader assessed a vertebral unit as 2 or 3, the patient was considered to have a syndesmophyte. The 2-reader agreement approach considered a patient to be mNY criteria-positive or having a syndesmophyte when at least 2 readers assessed the patient as such.

Inter-rater reliability was calculated by intraclass correlation coefficient (ICC), based on 2 reading scores made on the same subject by the primary readers. The smallest detectable change (SDC) was calculated using the Bland–Altman analysis at an 80% level of agreement (LoA) based on the data from the 2 primary readers [18].

The full analysis set from week 104 database lock (final reading campaign) was used for the analysis. Data from the secukinumab loading and non-loading dose groups were pooled for this imaging post hoc analysis. Data are presented as observed.

For radiographs, data are presented according to their original assignment at randomization with patients who switched to standard of care included as originally randomized and placebo patients who switched to secukinumab included in the placebo-secukinumab group. Patients who switched from blinded to open-label secukinumab 150 mg were included and presented as originally randomized (secukinumab group). For MRI, patients originally randomized to placebo who switched to active treatment were not included in the analysis. Patients randomized to secukinumab 150 mg blinded who switched to open-label secukinumab 150 mg were included and presented as originally randomized.

## Results

A total of 438 of the 555 patients (78.9%) completed week 104 of the study. Overall, 50.8% of the patients in the loading dose group, 47.3% in the non-loading dose group, and 64% in the placebo group switched to either open-label secukinumab or standard of care (only 3 patients switched to standard of care) between weeks 20 and 52. Starting at week 52, all patients (except for 3 patients who had switched to standard of care) received open-label secukinumab 150 mg treatment. Thus, all patients received secukinumab treatment for at least 1 year during the 104-week study period. Demographic and baseline disease characteristics were comparable across treatment groups [14]. Table 1 shows the imaging baseline characteristics in the patients.

**Table 1:** Baseline imaging characteristics

Variable	Secukinumab 150 mg with loading (*n* = 185)	Secukinumab 150 mg without loading (*n* = 184)	Secukinumab pooled (*n* = 369)	Placebo (*n* = 186)
Radiographs				
Total sacroiliitis score, mean (SD) (range, 0–8)	1.40 (1.47)	1.44 (1.50)	1.42 (1.48)	1.51 (1.58)
mSASSS score, mean (SD) (range, 0–72)	0.80 (2.70)	0.75 (2.59)	0.77 (2.64)	0.84 (2.43)
Patients with syndesmophytes, *n* (%)	29 (15.7)	33 (17.9)	62 (16.8)	27 (14.7)
MRI				
SI joint bone marrow edema score, mean (SD) (0–24)	2.14 (3.52)	1.94 (3.39)	2.04 (3.45)	2.26 (3.69)
Spinal inflammation, Berlin score, mean (SD) (0–69)	0.78 (1.70)	0.86 (1.81)	0.82 (1.75)	1.07 (2.28)

All patients with baseline assessments are included

*MRI* magnetic resonance imaging, *mSASSS* modified Stoke Ankylosing Spondylitis Spine Score, *SD* standard deviation, *SI* sacroiliac

### Radiographs

#### Sacroiliac joints

A total of 277 patients (75.1%) in the pooled secukinumab group and 139 (74.7%) in the placebo-secukinumab group had SI joint radiographs evaluable for change in total sacroiliitis score (i.e., had values available for both baseline and week 104). The mean (standard deviation, SD) baseline total sacroiliitis scores (range, 0–8) in the secukinumab and placebo-secukinumab groups were 1.45 (1.53) and 1.47 (1.60), respectively. At week 104, the scores were 1.41 (1.47) and 1.50 (1.61) in the secukinumab and placebo-secukinumab groups, respectively. Thus, only minimal changes in total sacroiliitis scores were observed over 2 years with a mean (SD) change of − 0.04 (0.49) in the secukinumab group and 0.04 (0.36) in the placebo-secukinumab group at week 104.

**Fig. 1:**
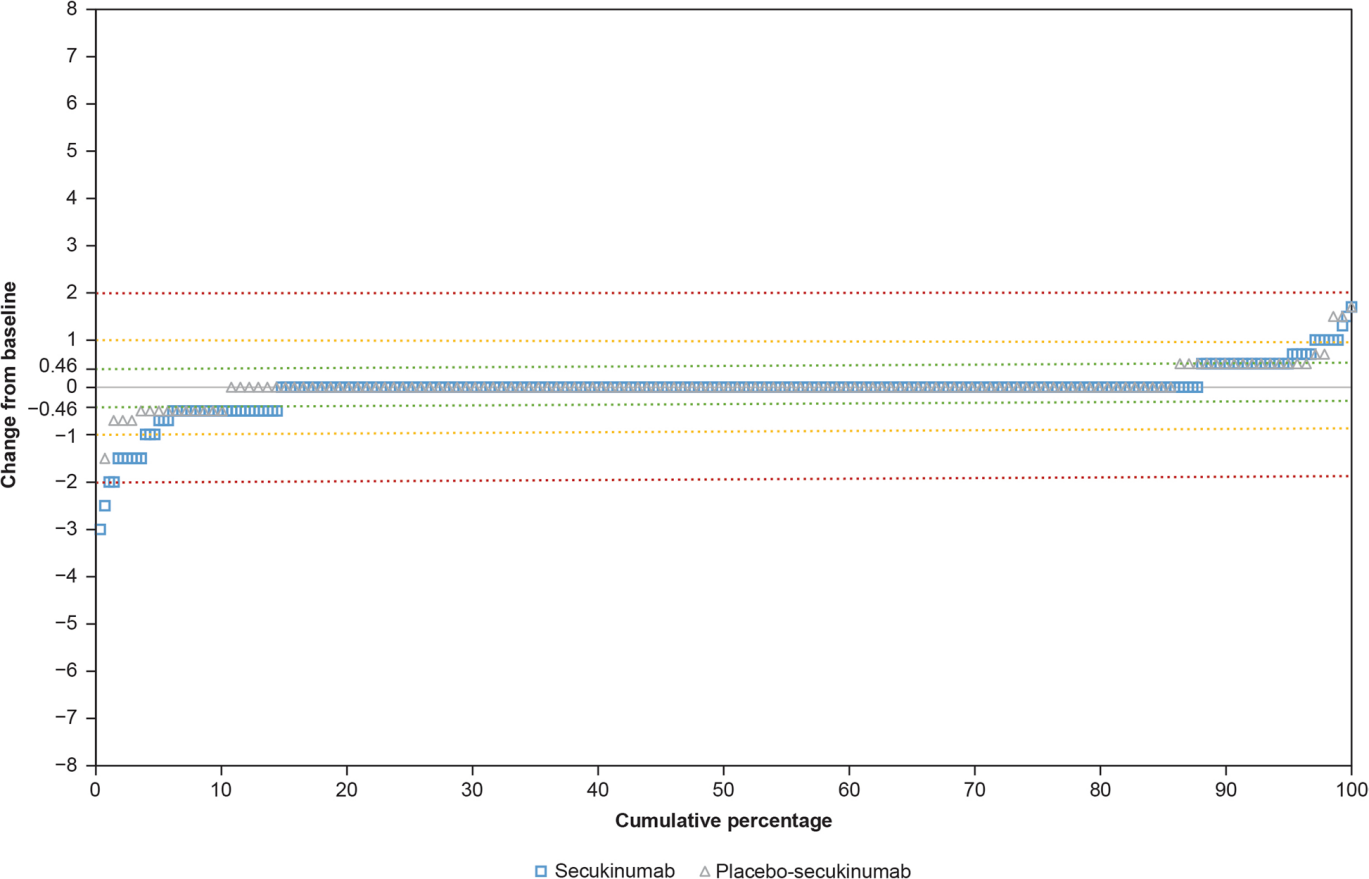
Cumulative probability of change from baseline to week 104 in SI joint total sacroiliitis score. Groups are presented as randomized (patients who switched to standard of care or from placebo to active treatment are analyzed according to the original treatment assignment at randomization). Green, yellow, and red lines represent the change from baseline in total sacroiliitis score of 0.46 (SDC), 1, and 2, respectively. SDC, smallest detectable change; SI, sacroiliac

Overall, 87.7% (243 of 277) of patients in the secukinumab group and 85.6% (119 of 139) of patients in the placebo-secukinumab group showed no progression in SI joint scoring by week 104. No progression was defined as an increase in total sacroiliitis score ≤ SDC, which was 0.46 at an 80% LoA. Clinically meaningful progression, defined as a total sacroiliitis score increase of > 1, was observed in only 1.1% (3 of 277) of patients in the secukinumab group and 2.2% (3 of 139) of patients in the placebo-secukinumab group. None of the patients had an increase of > 2 in the total sacroiliitis score (Fig. 1). Overall, the number of patients with clinically meaningful progression was too low to determine if patients with definite SI joint inflammation on MRI at baseline (SI joint BME > 2) were more likely to have a total sacroiliitis score increase > 1 over 2 years than those with lesser SI joint inflammation on MRI (SI joint BME ≤ 2). ICC for inter-rater reliability of paired total sacroiliitis score was 0.59 and 0.57 at screening and week 104, respectively, and ICC for change from screening at week 104 was 0.29.

Patients whose screening SI joint radiographs fulfilled the mNY criteria during the eligibility reading session were excluded from participation in the study. When screening radiographs of eligible patients were scored alongside post-baseline images in the final reading campaign, 24.5% of patients in either group (68 of 277 and 34 of 139 patients in the secukinumab and placebo-secukinumab groups, respectively) were evaluated as mNY criteria-positive on radiographs obtained at screening, by at least 1 reader. Of these patients, 16.2% (11 of 68) in the secukinumab group and 14.7% (5 of 34) in the placebo-secukinumab group were evaluated as mNY-negative at week 104 (Table 2). Among patients whose screening radiographs were mNY-negative, 96.7% (202 of 209) and 97.1% (102 of 105) of patients in the secukinumab and placebo-secukinumab groups, respectively, stayed negative through week 104. Only 7 patients (3.3%) in the secukinumab group and 3 (2.9%) in the placebo-secukinumab group who were mNY-negative at baseline were scored as mNY-positive at week 104 (Table 2). Thus, in both treatment groups, fewer patients progressed from mNY-negative to mNY-positive than had a change in the opposite direction (from positive to negative) resulting in an overall negative net progression. When the 2-reader agreement was considered, the rates of patients with baseline radiographs scored as mNY-positive in the final reading campaign were lower vs single-reader assessment (7.9% [22 of 277] in the secukinumab group and 12.9% [18 of 139] in the placebo-secukinumab group vs 24.5% in either group based on single reader assessment). The net progression remained negative with a higher proportion of patients in either group changing from positive to negative (18.2% [4 of 22] and 22.2% [4 of 18] in the secukinumab and placebo-secukinumab groups, respectively) than those progressing from negative to positive (1.2% [3 of 255] and 0.0% [0 of 121] in secukinumab and placebo-secukinumab groups, respectively) (Table 2). This negative net progression likely reflects an inherent variability of mNY criteria assessment rather than a “healing” process.

**Table 2:** mNY status at screening and at week 104

		**Screening**	**Week 104**	
			**mNY-negative**	**mNY-positive**
**Single-reader assessment**^**a**^	**Secukinumab (*N* = 277)**	mNY-negative, *n* = 209	202/209 (96.7%)	7/209 (3.3%)
		mNY-positive, *n* = 68	11/68 (16.2%)	57/68 (83.8%)
	**Placebo-secukinumab (*N* = 139)**	mNY-negative, *n* = 105	102/105 (97.1%)	3/105 (2.9%)
		mNY-positive, *n* = 34	5/34 (14.7%)	29/34 (85.3%)
**Two-reader agreement**^**b**^	**Secukinumab (*N* = 277)**	mNY-negative, *n* = 255	252/255 (98.8%)	3/255 (1.2%)
		mNY-positive, *n* = 22	4/22 (18.2%)	18/22 (81.8%)
	**Placebo-secukinumab (*N* = 139)**	mNY-negative, *n* = 121	121/121 (100.0%)	0/121 (0.0%)
		mNY-positive, *n* = 18	4/18 (22.2%)	14/18 (77.8%)

SI joint grade scoring was performed by 2 independent readers (or 3 if adjudicated) who were blinded to the image sequence and treatment assignment. Only patients with both baseline and week 104 assessments are included

*mNY* modified New York, *SI* sacroiliac

^a^A patient was counted as mNY-positive if considered mNY-positive according to at least 1 reader

^b^A patient is counted as mNY-positive if considered mNY-positive according to at least 2 readers

#### Spine

A total of 280 patients in the pooled secukinumab group and 136 in the placebo-secukinumab group had spine radiographs evaluable for change in mSASSS score (i.e., had values available for both baseline and week 104). At baseline, the mean (SD) mSASSS scores in the secukinumab and placebo-secukinumab groups were 0.68 (2.35) and 0.81 (2.37), respectively. At week 104, the scores were 0.73 (2.49) and 0.88 (2.60) in the secukinumab and placebo-secukinumab groups, respectively. Thus, there was a minimal change over 2 years with a mean (SD) change of 0.04 (0.47) in the secukinumab group and 0.07 (0.36) in the placebo-secukinumab group at week 104.

**Fig. 2:**
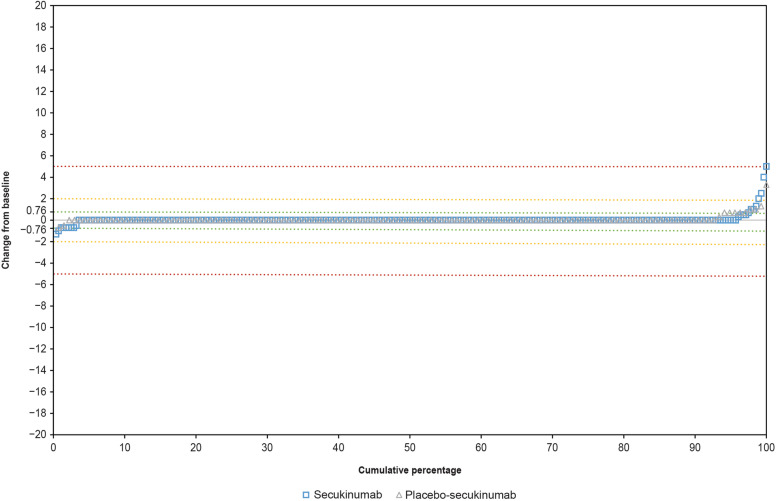
Cumulative probability of change from baseline to week 104 in mSASSS total score. Groups are presented as randomized (patients who switched to standard of care or from placebo to active treatment are analyzed according to the original treatment assignment at randomization). Green, yellow, and red lines represent the change from baseline in total mSASSS score of 0.76 (SDC), 2, and 5, respectively. SDC, smallest detectable change; mSASSS, modified Stoke Ankylosing Spondylitis Spine Score

Overall, 97.5% (273 of 280) of patients in the secukinumab group and 97.1% (132 of 136) in the placebo-secukinumab group showed no structural progression over 2 years, when no progression was defined as a change in total mSASSS score ≤ SDC (which was 0.76 at 80% LoA). An increase of > 2 units in the mSASSS total score was observed in 1.1% (3 of 280) of patients in the secukinumab group and 0.7% (1 of 136) in the placebo-secukinumab group. None of the patients had an increase of > 5 (Fig. 2). ICC for inter-rater reliability of paired mSASSS score was 0.70 and 0.66 at screening and week 104, respectively. Due to the majority of images indicating no change from screening to week 104, the estimated covariance matrix for random effect was 0, rendering the model unreliable. Therefore, the ICC for change from screening was not meaningful.

When a syndesmophyte scored by at least 1 reader was considered, most of the patients (84.6% [237 of 280] in the secukinumab group and 86.0% [117 of 136] in the placebo-secukinumab group) had no syndesmophytes at baseline. Among these, 1.7% (4 of 237) in the secukinumab group and 3.4% (4 of 117) in the placebo-secukinumab group developed at least 1 new syndesmophyte (at least 1 vertebral corner assigned a score of ≥ 2 by at least 1 reader) over 2 years. On the other hand, among patients reported to have at least 1 syndesmophyte at baseline (15.4% of patients [43 of 280] in the secukinumab group and 14.0% of patients [19 of 136] in the placebo-secukinumab group), a higher proportion of patients, i.e., 20.9% (9 of 43) in the secukinumab group and 36.8% (7 of 19) in the placebo-secukinumab group, had developed at least 1 new syndesmophyte by week 104. Also, irrespective of the treatment group, the majority of patients (79% based on an assessment by a single reader) who had at least 1 syndesmophyte at screening were mNY-negative at baseline (Additional file 1: Table S1).

When the 2-reader agreement was considered, fewer patients had syndesmophytes at baseline (4.6% [13 of 280] in the secukinumab group and 8.8% [12 of 136] in the placebo-secukinumab group), and a lower proportion of these patients developed new syndesmophytes (15.4% [2 of 13] in the secukinumab group and 8.3% [1 of 12] in the placebo-secukinumab group) compared with the single-reader assessment. Among patients without syndesmophytes at baseline, only 1 patient (in the secukinumab group) had developed a syndesmophyte at week 104. Importantly, the proportion of patients who had a syndesmophyte at baseline and were considered mNY-negative was even higher when both evaluations required a 2-reader agreement (88% vs 79% based on single reader assessment) (Additional file 1: Table S1).

### MRI

#### Sacroiliac joints

**Fig. 3:**
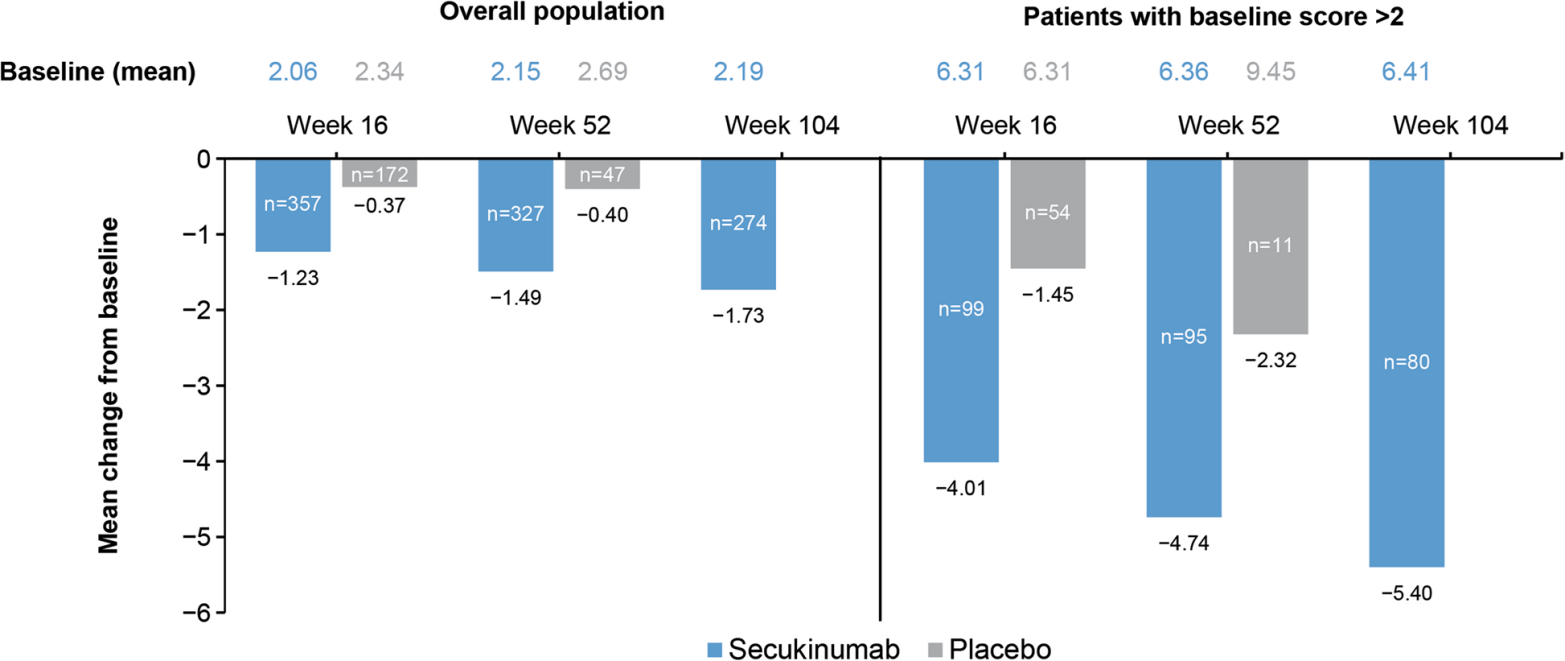
The mean change in SI joint bone marrow edema score by MRI in the overall population and in patients with baseline score > 2 through week 104. Data presented are as observed. The secukinumab group includes patients who continued on secukinumab to week 104. The placebo group includes only patients randomized to placebo who remained on placebo to week 52. At each time point, only patients with a value at both baseline and that time point are included. MRI, magnetic resonance imaging; *n*, number of evaluable patients; SI, sacroiliac

At week 16, the mean (SD) SI joint total edema score decreased from baseline by 1.23 (2.81) in the secukinumab group vs 0.37 (1.90) in the placebo group. This reduction in BME observed at week 16 was sustained through week 104 (−1.49 [3.33] with secukinumab vs −0.40 [2.28] with placebo at week 52; −1.73 [3.49] with secukinumab at week 104) (Fig. 3). A similar trend was observed in patients with a complete set of images (images available at all 4 time points; sensitivity analysis) (Additional file 1: Fig. S2). As expected, a greater reduction was seen in patients with definite SI joint inflammation at baseline defined as a baseline score of > 2 (mean reduction in score by 4.01, 4.74, and 5.40 at weeks 16, 52, and 104 respectively, in the secukinumab group) (Fig. 3). The ICC for inter-rater reliability of paired SI joint total edema score change from screening was 0.79, 0.88, and 0.90 at weeks 16, 52, and 104, respectively.

#### Spine

Spinal inflammation on MRI (Berlin score) was low at baseline across the groups. The mean score in the secukinumab group was 0.82, and it was 1.07 in the placebo group. The mean score at week 104 in the secukinumab group was 0.56.

## Discussion

This exploratory analysis from the PREVENT study evaluated radiographic changes and spinal inflammation in patients with active nr-axSpA over a period of 2 years. This is one of the largest imaging datasets in patients with nr-axSpA available to date. The overall radiographic changes, along with any changes in spinal inflammation, in a population of patients on an effective biologic therapy were minimal over time, while a reduction in SI joint inflammation was observed as previously reported [14].

Most of the patients in the secukinumab group (87.7%), as well as those originally randomized to the placebo group (85.6%), did not have an increase in total sacroiliitis score above the SDC on SI joint radiographs, indicating no radiographic progression over 2 years. The SDC for the total sacroiliitis score was 0.46. Notably, given that the minimum possible change on mNY grading scale is 1, the smallest change that can be observed in an individual patient when considering the average score by 2 readers is 0.5. None of the patients in either group had an increase in total sacroiliitis score of > 2, and only few had an increase of > 1 (1.1% in the secukinumab group and 2.2% in the placebo group). Furthermore, the proportion of patients who had an increase in the total sacroiliitis score greater than the SDC was similar to the proportion of patients who had a decrease in the score of similar magnitude, suggesting that the observed change may be attributed to reader variability rather than disease progression.

As per the eligibility criteria, any patient with mNY-positive status at screening based on the eligibility read by a single reader was excluded from the study. However, when the same images from screening were scored again alongside post-baseline images at week 104, by readers blinded to the sequence of imaging, some of the radiographs were scored as mNY-positive. The poor-to-moderate intra-rater and inter-rater reliability of SI joint damage assessment according to the mNY criteria are well described [19–21]. Cases of apparent structural “improvement” in SI joints are found even when images are evaluated by highly trained central readers, especially when readers are blinded to the image sequence. An overall net change approach accounts for measurement error in the interpretation of radiographic damage over time [22]. Here, despite the measurement error, about 97% of the patients in either group stayed mNY-negative through 2 years and with fewer patients changing from negative to positive (compared with those changing from positive to negative), there was a negative net change. This negative change is likely a reflection of inherent variability and limited progression rather than true improvement. With the 2-reader agreement, while the number of patients considered positive at screening dropped, the overall net progression remained negative, underscoring the negligible progression in SI joint abnormalities in the study population over 2 years. The generally very low level of structural changes at baseline in a nr-axSpA population renders the radiographic evaluation more susceptible to reading variability. Given that high rates of reader variability are commonly seen with SI joint radiograph evaluation, an artificial and sharp cutoff of the mNY criteria may not serve as the best tool to capture progression.

While earlier studies suggested low but evident progression in early axSpA, more recent studies report even lower rates of radiographic SI joint structural progression. In the RAPID-axSpA study, limited changes in SI joint grading were observed after 4 years in patients treated with certolizumab: 4.5% (2 of 44) of patients who were mNY-negative at baseline fulfilled the mNY criteria at week 204 and 4.3% (4 of 93) of patients who were initially mNY-positive were evaluated as mNY-negative at week 204 [23]. There are a limited number of randomized controlled studies evaluating the progression in nr-axSpA. However, there is some evidence from observational cohorts. Observations from a recent prospective Esperanza cohort (2020) showed that 16 of 94 analyzed patients changed sacroiliitis status over 6 years: 7 patients changed from baseline mNY-negative to mNY-positive, and 9 patients changed from baseline mNY-positive to mNY-negative [24]. The net change in total sacroiliitis score over a period of 6 years was − 0.25 [24] compared to − 0.04 in the PREVENT study over a period of 2 years. On the other hand, Dougados et al. reported a net progression from nr-axSpA to radiographic axSpA of 5.1% at 5 years (*N* = 416) in the DEvenir des Spondyloarthrites Indifférenciées Récentes (DESIR) cohort (prevalence cohort of early axSpA, < 3 years duration) [25]. However, the difference in patient population (broad vs controlled with specific inclusion/exclusion criteria including duration of disease, etc.) and study design (particularly treatment, which may not be controlled or defined in a cohort study) should be considered while comparing results from cohorts to randomized studies.

Lower rates of progression reported in more recent studies may reflect greater awareness of the measurement error in radiograph scoring leading to the implementation of central reading with more than 2 readers and also acknowledging the measurement error in the analysis. Another potential reason for low to negligible progression rates reported here and in other recent studies could be the nr-axSpA population recruited into these clinical trials, who may present with less severe disease and have earlier access to biologic treatment.

With regard to the mSASSS score, most patients in both groups showed no progression to week 104. While none of the patients in either group had an mSASSS total score increase of > 5, around 1% had an increase of > 2. The mean change in mSASSS score was 0.04 in the secukinumab group (0.68 at baseline). Previous studies have similarly reported limited spinal progression in patients with nr-axSpA. In the RAPID-axSpA study, which reported 4-year imaging outcomes, the mean mSASSS change was − 0.01 (95% confidence interval [CI], − 0.19 to 0.17) from baseline to week 96 and 0.06 (95% CI, − 0.17 to 0.28) to week 204 in patients with nr-axSpA treated with certolizumab pegol [23]. Progression (defined as an mSASSS increase of ≥ 2 points) was observed in 2 of 141 patients with nr-axSpA [23]. In the Swiss Clinical Quality Management cohort, the mean (SD) spinal progression in patients with nr-axSpA (with a mean disease duration of 10 years) was 0.16 (0.62) mSASSS units over 2 years [26]. In the DESIR cohort, the mean (SD) mSASSS progression was 0.2 (0.9) at 2 years and 0.4 (1.8) at 5 years [27]. The results from clinical studies have shown the progression of spinal damage to be very limited in this patient population thus suggesting that radiographic progression may not be a highly valuable outcome measure in patients with nr-axSpA, at least not over a relatively short duration of 2 years.

In the present study, approximately 15% of patients had syndesmophytes at baseline identified by at least 1 reader (6% when reader agreement was required). Similarly, 20% of the nr-axSpA patients in the RAPID-axSpA study, 9% of patients in the Swiss Clinical Quality Management cohort (readers’ agreement was required in both studies), and 7% of patients in the DESIR cohort (2 of the 3 readers were required to identify the syndesmophyte) had syndesmophytes at baseline, suggesting that the process of structural damage starts early in the disease at least in some patients [23, 26, 27]. The presence of syndesmophytes at baseline has been consistently found to be the strongest predictor of the development of new syndesmophytes and radiographic progression [28–34]. Similarly in this study, in either group, the proportion of patients who had developed new syndesmophyte(s) by 2 years was higher among patients with at least 1 syndesmophyte at baseline, than among patients who had none at baseline.

Syndesmophyte formation is thought to be mainly associated with r-axSpA [26]. However, in this study, the majority of patients who presented with a syndesmophyte at baseline were classified as mNY criteria negative by experienced readers (79% by single reader assessment or 88% when reader agreement was required). Minor changes in pelvic radiographs in early disease are particularly challenging to score (grade 1 versus grade 2 sacroiliitis), resulting in limited reliability of the mNY criteria to discriminate between patients with non-radiographic versus radiographic disease [35]. Findings from conventional radiography of the spine, including the presence of syndesmophytes, are not taken into consideration for the purpose of classification criteria defining the non-radiographic patient population in clinical trials. A considerable proportion of patients with spinal structural damage as evidenced by the presence of syndesmophytes yet no definitive SI joint changes (meeting mNY criteria) may be classified as non-radiographic contributing to the heterogeneity of the nr-axSpA population in the research setting. These results, along with previous data reported by others, question the term nr-axSpA which may be misleading. The authors of this study agree that the term should not be used for diagnosis but only for the classification of patients for clinical trials. For diagnosis, the term axSpA is preferred [36].

This study had some limitations. Patients were allowed to switch to open-label secukinumab or standard of care leading to a lack of placebo control after 20 weeks. The analyses of images were conducted on patients who continued on the study, thus were likely to respond well to the treatment introducing a potential bias. Also, in this study, inter-rater reliability was modest at best. However, this is not surprising in sacroiliitis assessment where low-to-moderate inter-rater/intra-rater reliability has been reported in previous studies [37, 38].

The value of minor effects of biological disease-modifying anti-rheumatic drugs (DMARDs) [39] or targeted synthetic DMARDs [40] on structural SI joint changes has been recently challenged [41]. In this study, with limited use of synthetic DMARDs, those effects were not assessed.

## Conclusions

In summary, in this large study of patients with nr-axSpA, the overall level of spinal inflammation or structural damage at baseline was low. Most patients initially randomized to secukinumab or placebo showed no radiographic progression in SI joints and spine over 2 years. Secukinumab reduced SI joint inflammation (BME) in patients with active nr-axSpA which was sustained over 2 years.

## Supplementary Information

**Additional file 1:** **Fig. S1.** Study Design. **Fig. S2.** Mean change in SI joint bone marrow edema score by MRI in the overall population and in patients with baseline score >2 through week 104 (in patients with images available at all 4 time points). **Table S1.** Syndesmophyte status at screening by mNY status at screening based on single reader assessment and two reader agreement. 

## Data Availability

The datasets generated and analyzed during the current study are not publicly available. Novartis is committed to sharing access to patient-level data and supporting clinical documents from eligible studies with qualified external researchers. These requests are reviewed and approved based on scientific merit. All data provided are anonymized to respect the privacy of patients who have participated in the trial, in line with applicable laws and regulations. Any requests should be made to the corresponding author.

## Abbreviations

AS: Ankylosing spondylitis
ASAS: Assessment of SpondyloArthritis International Society
ASspiMRI: Ankylosing spondylitis spine magnetic resonance imaging
BME: Bone marrow edema
CRP: C-reactive protein
ICC: Intraclass correlation coefficient
LoA: Level of agreement
mNY: modified New York
mSASSS: modified Stoke Ankylosing Spondylitis Spine Score
MRI: Magnetic resonance imaging
nr-axSpA: Non-radiographic axial spondyloarthritis
r-axSpA: Radiographic axial spondyloarthritis
SD: Standard deviation
SDC: Smallest detectable change
SI: Sacroiliac
