# Supplementary material for: Two-year imaging outcomes from a phase 3 randomized trial of secukinumab in patients with non-radiographic axial spondyloarthritis
Source: Arthritis Res Ther. 2023 May 16;25:80. doi: 10.1186/s13075-023-03051-5 (PMC10186767; doi:10.1186/s13075-023-03051-5)
Supplement: Supplementary file 1 — Additional file 1: Fig. S1. Study Design. Fig. S2. Mean change in SI joint bone marrow edema score by MRI in the overall population and in patients with baseline score >2 through week 104 (in patients with images available at all 4 time points). Table S1. Syndesmophyte status at screening by mNY status at screening based on single reader assessment and two reader agreement. [file 13075_2023_3051_MOESM1_ESM.docx]

**Supplementary appendix**

**Table of Contents**

|  | **Page no.** |
| --- | --- |
| Figure S1: Study Design | 2 |
| Figure S2: Mean change in SI joint bone marrow edema score by MRI in the overall population and in patients with baseline score >2 through week 104 (in patients with images available at all 4 time points) | 3 |
| Table S1. Syndesmophyte status at screening by mNY status at screening based on single reader assessment and two reader agreement | 4 |

**Figure S1: Study design**

**
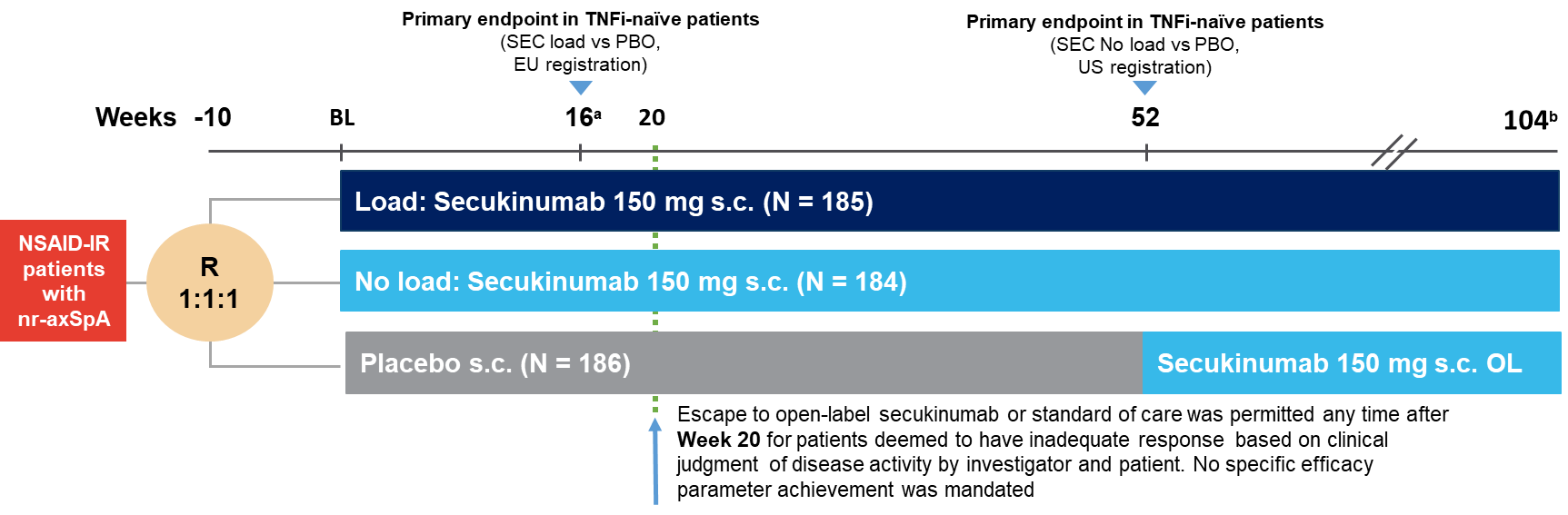
**

^a^Treatment modifications (NSAIDs, corticosteroids, and DMARDs) to treat signs/symptoms of nr-axSpA were allowed only after week 16. ^b^At week 104, all patients who finish the core phase per protocol on study medication could join the extension phase of the study.

BL, baseline; DMARDs, disease modifying anti-rheumatic drugs; N, total number of randomized patients; NSAIDs, non-steroidal anti-inflammatory drugs; nr-axSpA, non-radiographic axial spondyloarthritis; OL, open-label; PBO, placebo; R, randomized; s.c., subcutaneous; SEC, secukinumab; SI, sacroiliac, TNFi, tumor necrosis factor inhibitor

Reproduced from: Deodhar A, Blanco R, Dokoupilova E, Hall S, Kameda H, Kivitz AJ, et al. Improvement of Signs and Symptoms of Nonradiographic Axial Spondyloarthritis in Patients Treated With Secukinumab: Primary Results of a Randomized, Placebo-Controlled Phase III Study. *Arthritis Rheumatol*. 2021;73(1):110-20. Supplementary Material. [Creative Commons — Attribution-NonCommercial-NoDerivatives 4.0 International — CC BY-NC-ND 4.0](https://creativecommons.org/licenses/by-nc-nd/4.0/)

**Figure S2: Mean change in SI joint bone marrow edema score by MRI in the overall population and in patients with baseline score >2 through week 104 (in patients with images available at all 4 time points)**


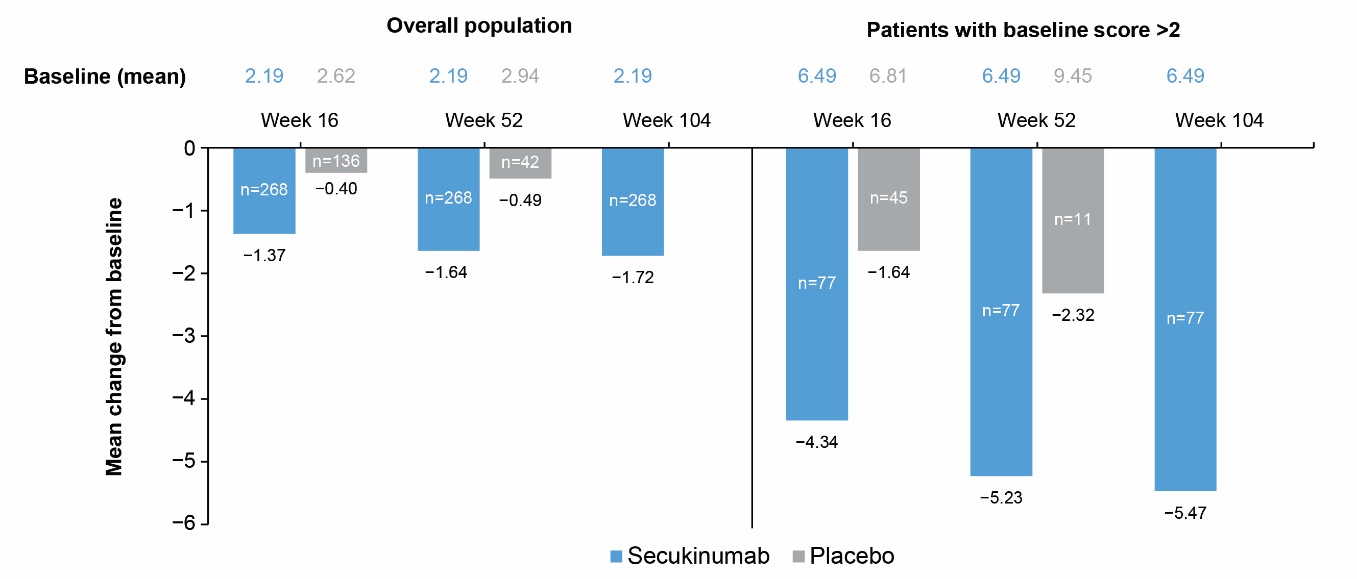


Data presented are as observed. Secukinumab group includes patients who continued on secukinumab to week 104. Placebo group includes only patients randomized to placebo who remained on placebo to week 52. At each time point, only patients with a value at both baseline and that time point are included.

MRI, magnetic resonance imaging; n, number of evaluable patients; SI, sacroiliac

**Table S1. Syndesmophyte status at screening by mNY status at screening based on single reader assessment and two reader agreement**

|  | | **mNY-positive** | **mNY-negative** |
| --- | --- | --- | --- |
| **Single reader assessment** | | | |
| Secukinumab | With syndesmophyte  n=43 | 8/43 (18.6%) | 35/43 (81.4%) |
|  | Without syndesmophyte  n=237 | 61/237 (25.7%) | 176/237 (74.3%) |
| Placebo-secukinumab | With syndesmophyte  n=19 | 5/19 (26.3%) | 14/19 (73.7%) |
|  | Without syndesmophyte  n=117 | 28/117 (23.9%) | 89/117 (76.1%) |
| Total | With syndesmophyte  n=62 | 13/62 (21.0%) | 49/62 (79.0%) |
|  | Without syndesmophyte  n=354 | 89/354 (25.1%) | 265/354 (74.9%) |
| **Two reader agreement** | | | |
| Secukinumab | With syndesmophyte  n=13 | 1/13 (7.7%) | 12/13 (92.3%) |
|  | Without syndesmophyte  n=267 | 21/267 (7.9%) | 246/267 (92.1%) |
| Placebo-secukinumab | With syndesmophyte  n=12 | 2/12 (16.7%) | 10/12 (83.3%) |
|  | Without syndesmophyte  n=124 | 15/124 (12.1%) | 109/124 (87.9%) |
| Total | With syndesmophyte  n=25 | 3/25 (12.0%) | 22/25 (88.0%) |
|  | Without syndesmophyte  n=391 | 36/391 (9.2%) | 355/391 (90.8%) |
| Only patients with both baseline and week 104 assessments for syndesmophyte and baseline assessment for mNY are included.  mNY, modified New York | | | |
